# Immunoregulation induced by autologous serum collected after acute exercise in obese men: a randomized cross-over trial

**DOI:** 10.1038/s41598-020-78750-z

**Published:** 2020-12-10

**Authors:** Gilson P. Dorneles, Igor M. da Silva, Maeli Andressa Santos, Viviane R. Elsner, Simone G. Fonseca, Alessandra Peres, Pedro R. T. Romão

**Affiliations:** 1 Laboratory of Cellular and Molecular Immunology, Department of Health Basic Sciences, Universidade Federal de Ciências da Saúde de Porto Alegre (UFCSPA), Rua Sarmento Leite, 245, Porto Alegre, RS 90050-170 Brazil; 2 Research Center, Methodist University Center IPA, Porto Alegre, RS 90420-060 Brazil; 3 Instituto de Patologia Tropical E Saúde Pública, Universidade Federal de Goiás, Goiânia, GO 74605-050 Brazil

**Keywords:** Physiology, Biomarkers, Diseases, Health care, Pathogenesis, Immunology, Cytokines, Inflammation, Lymphocytes

## Abstract

In this study, we evaluated the effects of autologous serum collected after two types of exercise on the in vitro inflammatory profile and T cell phenotype of resting peripheral blood mononuclear cells (PBMCs) in obese men. Serum samples and PBMCs were obtained from eight obese men who performed two exercise bouts—high intensity interval exercise (HIIE) and exhaustive exercise session to voluntary fatigue—in a randomized cross-over trial. Pre-exercise PBMCs were incubated with 50% autologous serum (collected before and after each exercise bout) for 4 h. In vitro experiments revealed that post-HIIE serum reduced the histone H4 acetylation status and NF-κB content of PBMCs and suppressed the production of both TNF-α and IL-6 by PBMCs, while increasing IL-10 production. Post-exhaustive exercise serum induced histone H4 hyperacetylation and mitochondrial depolarization in lymphocytes and increased TNF-α production. In vitro post-HIIE serum incubation resulted in an increase in the frequencies of CD4 + CTLA-4 + and CD4 + CD25+ T cells expressing CD39 and CD73. Post-exhaustive exercise serum decreased the frequency of CD4 + CD25 + CD73+ T cells but increased CD4 + CD25-CD39 + T cell frequency. Both post-exercise serums increased the proportions of CD4 + PD-1 + and CD8 + PD-1+ T cells. Blood serum factors released during exercise altered the immune response and T cell phenotype. The type of exercise impacted the immunomodulatory activity of the post-exercise serum on PBMCs.

## Introduction

Acute endurance exercise elicits a transient increase in the number of peripheral blood mononuclear cells (PBMCs), including T cells^1^. Typically, 70–80% of the mononuclear cells in blood are lymphocytes, which are the main players in the coordination of adaptive immune response^2^. An augmentation in the number of PBMCs occurs immediately after exercise, mainly due to the increased peripheral frequencies of CD4 + and CD8+ T cells and natural killer (NK) cells^1,3^. The T cell phenotype is widely influenced by exercise, with a preferential mobilization of memory effector T cells and immunoregulatory cells (i.e., regulatory T cells, Treg cells)^4,5^. In this sense, exercise induces an increase in the peripheral frequency of senescent T cells (CD4 + and CD8 + cells that are CD28-), effector memory CD4 + CD25-CD39+ T cells, and CD4 + CD25 + CD39 + memory Treg (mTreg) cells^6,7^. Furthermore, cytokine production by PBMCs (mainly by monocytes, lymphocytes, and NKs) changes in response to exercise^8,9^.

In recent years, participation of the purinergic system in the regulation of lymphocyte phenotype and function has been observed. The CD4 + CD25+ T cells metabolize adenosine triphosphate (ATP) into adenosine to induce immunomodulatory responses, mainly through Treg cells^10,11^. Ectonucleoside triphosphate diphosphohydrolase‐1 (ENTPD1, CD39) is an ectoenzyme, expressed in several cells including leukocytes, that hydrolyzes ATP and adenosine diphosphate (ADP) to adenosine monophosphate (AMP). Subsequently, ecto‐5′‐nucleotidase (CD73) converts AMP to adenosine, which is a well‐known immunosuppressive metabolite^12,13^. Furthermore, CD4+ T cells expressing the surface marker cytotoxic T-lymphocyte-associated protein 4 (CTLA-4) have strong inhibitory effects on other leukocytes through cell-to-cell interactions^14^. Moreover, both CD4 + and CD8+ T cells expressing the programmed cell death protein 1 (PD-1) downregulate the immune response and promote self-tolerance by suppressing T cell function^15^. In this regard, the increased expression of ectonucleotidases, CTLA-4, and PD-1 is associated with an immunoregulatory, suppressive, and anti-inflammatory profile^16^.

Modifications in the cell phenotype are also linked to their functional activity. Post-translational histone acetylation-deacetylation events play a pivotal role in chromatin accessibility regulation, promotion of nuclear factor kappa B (NF-κB) recruitment, and increased pro-inflammatory cytokine gene transcription^17^. Acetylation of histone H4 (H4ac) is especially important for the chromatin structure, gene expression, cell polarization, and cytokine production by myeloid cells^18,19^. Hence, the activation of NF-κB and production of tumor necrosis factor-alpha (TNF-α) and interleukin (IL)-8 are widely dependent on the H4ac status^17^. Moreover, chromatin remodeling through histone acetylation impacts the immunoregulatory molecules expressed on the cell surface of lymphocytes, such as CTLA-4, CD39, and CD73 in CD4+ T cells, and histone acetyltransferase overexpression increases PD-1 expression in lymphoid cells^20,21^.

The acute systemic physiological response to exercise involves a wide range of metabolic, biochemical, immunological, and hormonal changes, wherein some mediators released into peripheral blood can have endocrine, autocrine, and paracrine effects in different cells and tissues, including immune sites^3^. One potential mechanism is that exercise alters systemic molecules (i.e. cytokines, extracellular vesicles, and metabolic factors) and proteins in the peripheral blood that influence the cytokine production by circulating mononuclear cells and the leukocyte phenotype^22,23^. It was demonstrated that acute exercise induces several systemic proteins, including apolipoproteins and immunological mediators, that may be involved in immunosurveillance during and after exercise bouts^24^. However, some exercise modalities, such as exhaustive exercise that induces higher degrees of muscle damage and systemic stress response, may induce a greater pro-inflammatory response and activation of peripheral leukocytes that become deregulated and detrimental to health^9,25^.

Different modes of exercise impact the T cell phenotype during and after exercise. Acute high-intensity exercise mobilizes Treg cells and T cells with activated and senescent phenotypes, with subsequent redistribution of these cells to inflamed exercised-tissues (i.e. muscle tissue)^5,6,26^. Furthermore, acute high-intensity, but not moderate-intensity exercise, induces a switch in intracellular transcription factor expression in T cells, favoring a type 2 inflammatory response mediated by the GATA3-IL-4 axis^27,28^. In contrast, exhaustive exercise increases intracellular NF-κB activation in peripheral lymphocytes from healthy men^29^. However, how the systemic factors released as a result of exercise (i.e. cytokines, hormones, and metabolic molecules) modulate the epigenetic events, inflammatory status, and phenotype of human circulating T cells and influence of the type and intensity of the bout in these immunoregulatory mechanisms, remains to be elucidated.

Conceptually, it is believed that exercise may mediate its effects on immune function by regulating systemic mediators released during each bout^30^. Chronic exercise training consists of frequent repetitions of acute bouts of exercise over time followed by recovery periods. In this regard, acute exercise and long-term exercise training represent two distinct situations with different physiological and immune alterations. The systemic physiological stress of acute exercise alters the blood composition and phenotype of immune cells by adjustments in cardiovascular parameters, neuroendocrine response, and metabolic dynamics^31^. In contrast, the effects of exercise training, i.e., improved fitness level, reduced adiposity, and enhanced muscle mass, can lead to the lowering of the circulating metabolic and inflammatory parameters that are recognized to alter PBMC phenotype and function^32^. While the adaptations to chronic exercise training and the physiological response to acute exercise have plausible mechanisms to improve immune function, so far, only a few studies have assessed the potential changes in blood serum composition induced by acute exercise concerning the immunological parameters^22,23,33,34^. Therefore, it is believed that intermediate metabolites released during exercise, such as lactate, may regulate the immune function during and after each bout. Incubation of PBMCs with lactate at concentrations similar to those occurring in circulation after moderate exercise (3.5 mM) reduced lipopolysaccharide (LPS)-induced inflammatory cytokine production^35^.

In fact, the blood composition directly affects the phenotype and the function of several cell lines^36–39^. Serum from humans under calorie restricted diets showed increased heat- and redox-induced stress resistance and upregulated genes related to longevity (i.e. Sirt1)^38,39^. Using Jurkat cells, an immortalized human T-lymphocyte cell line, Radom-Aizik et al.^34^ reported that the incubation of 10% of the serum from healthy men obtained 30 min after high-intensity exercise (80% of the individual peak work rate) resulted in the suppression of IL-2 and TNF-α production by those cells. As such, our study evaluated the impact of autologous serum collected after two types of exercise on cytokine production, NF-κB activity, epigenetic markers (H3ac and H4ac status), redox state of PBMCs, and changes in the phenotype of circulating CD4 + and CD8+ T cells of obese men. We hypothesized that a) the incubation of resting PBMCs from obese individuals with autologous serum collected after exercise results in changes in the T cell phenotype and cytokine production, epigenetic markers, and the NF-κB content and b) an immunoregulatory profile of PBMCs would be induced by autologous serum collected after high-intensity interval exercise (HIIE).

## Results

### Experimental study design

We performed a two-phase study to address the study aim. One week after the preliminary trial, participants engaged in the first study phase: two isocaloric exercise trials with a one-week interval between. The HIIE session consisted of a warm‐up (5 min) at a workload eliciting 40% maximum velocity (MAV) followed by 10 bouts of 60 s (85–90% MAV) intercepted by 75 s of active recovery (50% MAV) on a motorized treadmill. The exhaustive exercise consisted of stepping up and down from a step. The stepping rhythm was paced acoustically at 60 beats per min with the same periods (1 s) for stepping up and down, respectively. When the participants were not able to maintain the required stepping rhythm, a 30 s recovery period was given. Blood samples were collected before and immediately after each exercise session to analyze blood biochemical markers, including systemic cytokines, hormones, and oxidative stress variables, and the peripheral frequency of the T cell phenotype.

**Figure 1:**
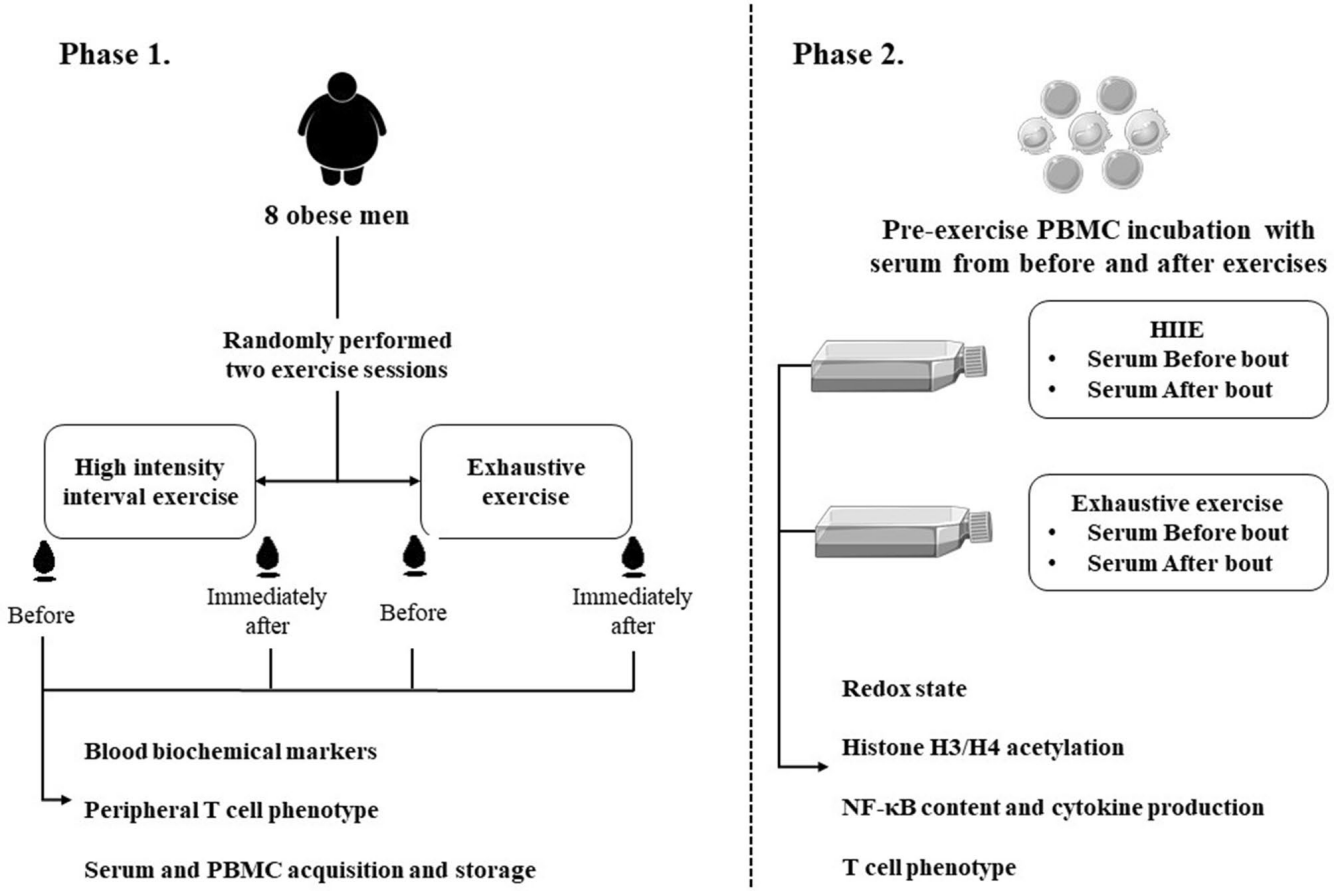
Study experimental design.

In phase 2 of the study, PBMCs acquired pre-exercise (“resting PBMCs”) from each participant were incubated with autologous serum (50%) collected before and after exercise bouts (HIIE and exhaustive) for 4 h in culture. Analyses of the cytokine production in the supernatant, T cell phenotype, histone H3/H4 acetylation status, NF-κB content, and redox state in PBMCs were conducted after the incubation time (Fig. 1).

### Physiological response to exercise and systemic serum biomarkers

Both protocols were well tolerated, and all participants completed all the bouts. There were no differences in energy expenditure between HIIE and exhaustive exercise (282 ± 14.2 kcal vs. 269 ± 20.01 kcal; *p* > 0.05). However, significant differences between groups were observed in relation to exercise duration (HIIE, 1800 ± 128 s; exhaustive exercise, 1232 ± 281 s; *p* < 0.001) and the average heart rate (HIIE, 148.18 ± 7.12 bpm; exhaustive exercise, 165.19 ± 5.01 bpm; *p* = 0.01).

Exercise significantly increased several serum biomarkers immediately after bouts (Table 1). Acute HIIE increased the systemic levels of IL-6 (*p* = 0.03; effect sizes (ES) = 1.13), IL-10 (*p* = 0.03; ES = 1.81), IGF-1 (*p* = 0.02; ES = 2.5), cortisol (*p* = 0.02; ES = 1.28), lactate (*p* < 0.001; ES = 4.01), uric acid (*p* = 0.04; ES = 1.59), and thiobarbituric acid reactive substances (TBARS) (*p* = 0.04; ES = 2.53) in the serum of the participants. A single bout of exhaustive exercise increased the serum concentrations of IL-6 (*p* = 0.03; ES = 2.18), TNF-α (*p* = 0.04; ES = 1.46), IL-17a (*p* = 0.04; ES = 0.74), IGF-1 (*p* = 0.01; ES = 2.29), cortisol (*p* = 0.02; ES = 1.69), lactate (*p* < 0.01; ES = 3.98), uric acid (*p* = 0.04; ES = 0.40), and TBARS (*p* < 0.01; ES = 4.24). Interestingly, TBARS levels were higher after exhaustive exercise compared to HIIE (*p* = 0.03; ES = 4.47). The serum leptin concentrations decreased immediately after HIIE (*p* = 0.03; ES = 0.94), without changes after exhaustive exercise (*p* > 0.05).

**Table 1:** The effect of HIIE and exhaustive exercise in serum biomarkers before and immediately after exercise.

	HIIE		Exhaustive exercise	
	Before	After	Before	After
IL-6 (pg/mL)	8.5 ± 2.8	10.9 ± 1.1^b^	8.3 ± 1.7	11.2 ± 0.8^b^
IL-10 (pg/mL)	12.3 ± 1.2	15.4 ± 2.1^b^	11.8 ± 2.1	10.9 ± 3.5
IL-17a (pg/mL)	7.9 ± 2.0	7.0 ± 3.8	7.5 ± 1.8	8.9 ± 2.0^b^
IFN-γ (pg/mL)	19.2 ± 1.3	18.5 ± 1.8	18.9 ± 2.0	19.4 ± 2.8
Leptin (ng/mL)	54.61 ± 8.1	47.10 ± 7.8^b^	53.1 ± 10.1	52.0 ± 8.1
TNF-α (pg/mL)	24.2 ± 4.9	25.1 ± 3.2	24.7 ± 3.5	28.9 ± 2.1^b^
IGF-1 (ng/mL)	123.1 ± 15.8	158.2 ± 11.2^b^	127.9 ± 13.9	168.6 ± 21.0^b^
Cortisol (µg/dL)	32.0 ± 3.9	36.0 ± 2.1^b^	31.5 ± 3.1	35.9 ± 2.0^b^
Glucose (mg/dL)	86.1 ± 15.1	83.0 ± 13.2	86.8 ± 8.0	85.2 ± 10.1
Lactate (mmol/L)	1.3 ± 0.6	7.5 ± 2.1^b^	1.2 ± 0.4	5.3 ± 1.4^b^
Uric acid (mg/dL)	5.3 ± 1.8	7.8 ± 1.3^b^	5.5 ± 2.1	6.3 ± 1.9^b^
TBARS (nmol/mL)	1.6 ± 0.2	2.0 ± 0.1^b^	1.5 ± 0.4	3.0 ± 0.3^a,b^

Data are presented as mean ± standard deviation.

^a^Denotes between groups difference (*p* < 0.05).

^b^Denotes statistical difference compared to before exercise (*p* < 0.05).

### Acute T cell mobilization in response to exercise bouts

Lymphocytosis was induced immediately after HIIE, characterized by increased peripheral frequencies of CD4 + and CD8+ T cells (*p* = 0.03, ES = 0.54; *p* < 0.001, ES = 0.88, respectively) (Table 2). Exhaustive exercise mobilized higher proportions of CD8+ T cells (*p* < 0.001; ES = 2.01), without difference in CD4 + T cell frequency (*p* > 0.05). The peripheral frequencies of CD4+ T cells expressing ectonucleotidases, CTLA-4, and PD-1 differentially changed in response to exercise protocols. Acute HIIE increased the peripheral frequency of CD4 + CD25-CD39 + (*p* = 0.03; ES = 0.79), CD4 + CD25 + CD39 + (*p* = 0.04; ES = 0.88), CD4 + CD25 + CD73 + (*p* = 0.04; ES = 0.36), CD4 + CTLA-4 + (*p* = 0.02; ES = 0.32), CD4 + PD-1 + (*p* = 0.03; ES = 0.78), and CD8 + PD-1 + (*p* = 0.03; ES = 0.70) without differences in the frequencies of CD4 + CD25-CD73 + and CD4 + CD25+ T cells co-expressing CD39 + CD73 + (*p* > 0.05). Conversely, acute exhaustive exercise increased the peripheral proportions of CD4 + CD25-CD39 + (*p* = 0.03; ES = 1.86), CD4 + PD-1 + (*p* = 0.03; ES = 0.39), and CD8 + PD-1 + (*p* = 0.04; ES = 1.0), but decreased the frequencies of CD4 + CD25-CD73 + (*p* = 0.02; ES = 1.68), CD4 + CD25 + CD73 + (*p* = 0.04; ES = 0.69), and CD4 + CTLA-4 (*p* = 0.04; ES = 0.38) (Table 2).

**Table 2:** The effect of HIIE and exhaustive exercise in peripheral frequency of T cells before and immediately after exercise.

	HIIE	Exhaustive exercise
**CD4 + (%)**		
Before	42.5 ± 6.5	41.6 ± 6.2
After	45.2 ± 2.9^b^	40.4 ± 8.3
**CD4 + CD25-CD39 + (%)**		
Before	11.0 ± 5.9	15.1 ± 1.4
After	14.8 ± 3.4^b^	18.0 ± 1.7^b^
**CD4 + CD25 + CD39 + (%)**		
Before	2.4 ± 0.5	1.8 ± 0.8
After	2.8 ± 0.4^b^	1.8 ± 0.9
**CD4 + CD25-CD73 + (%)**		
Before	22.0 ± 7.1	21.1 ± 3.9
After	20.5 ± 3.5	14.8 ± 3.6^b^
**CD4 + CD25 + CD73 + (%)**		
Before	2.3 ± 1.7	1.6 ± 0.9
After	2.9 ± 1.6^b^	1.1 ± 0.5^b^
**CD4 + CD25 + CD39 + CD73 + (%)**		
Before	11.5 ± 4.4	11.7 ± 2.8
After	14.5 ± 7.9	12.1 ± 3.0
**CD4 + CTLA-4 + (%)**		
Before	6.5 ± 2.0	10.2 ± 6.4
After	7.2 ± 2.3^b^	8.1 ± 4.5^b^
**CD4 + PD-1 + (%)**		
Before	3.6 ± 1.8	2.9 ± 1.4
After	5.0 ± 1.8^b^	3.5 ± 1.7^b^
**CD8 + (%)**		
Before	22.7 ± 3.7	21.7 ± 4.6
After	27.2 ± 6.2^b^	28.9 ± 2.1^b^
**CD8 + PD-1 + (%)**		
Before	1.1 ± 0.4	1.3 ± 0.9
After	1.5 ± 0.7^b^	2.3 ± 1.1^b^

Data presented as mean ± standard deviation.

^a^Denotes statistical difference compared to before exercise (*p* < 0.05).

^b^Denotes statistical difference compared to before exercise (*p* < 0.05).

### Autologous serum collected after exercise bouts changes the redox state and inflammatory profile of PBMCs

**Figure 2:**
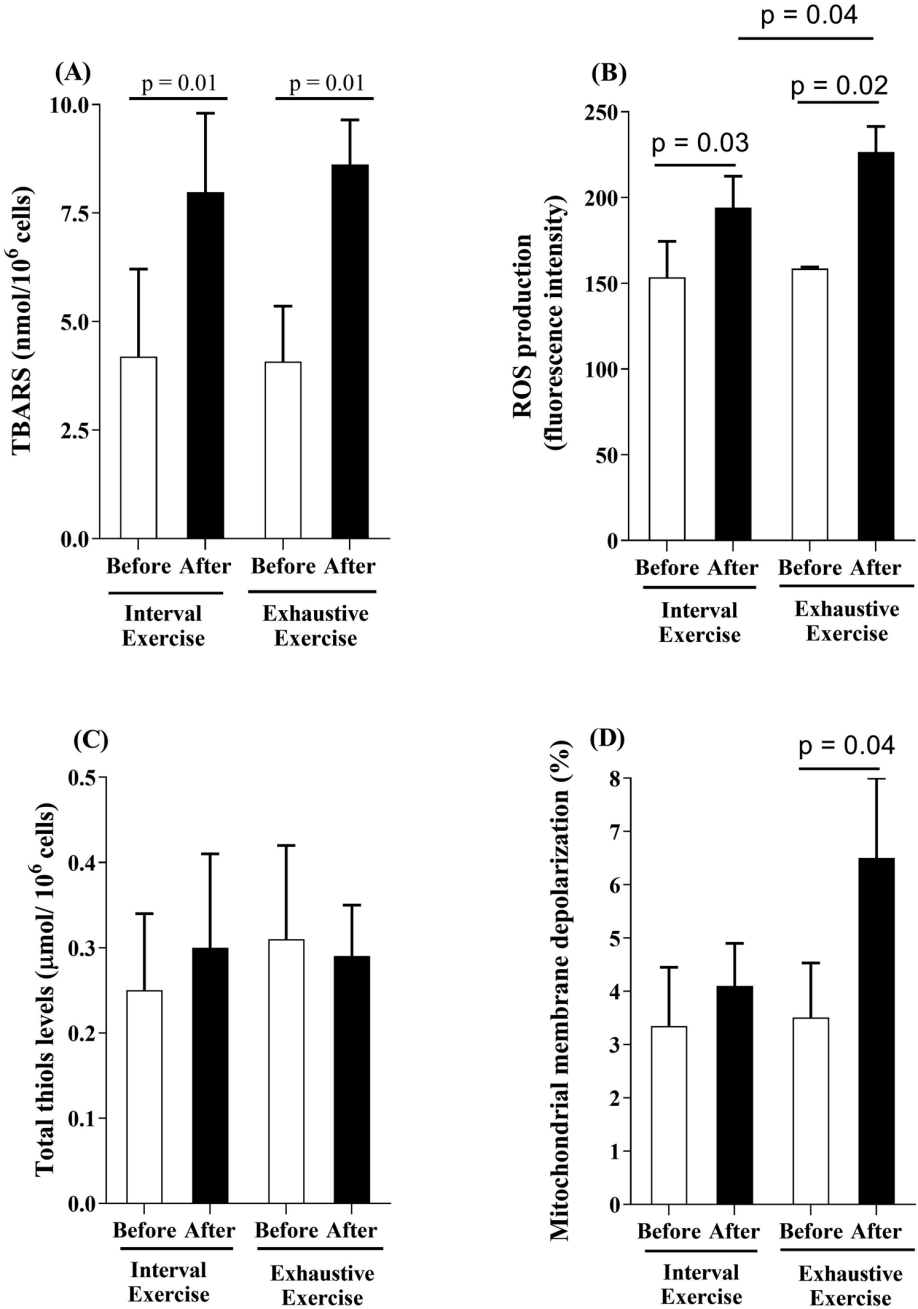
The redox state of mononuclear cells incubated with autologous serum from before (white bars) and after (black bars) two types of exercise. Lipid peroxidation (**A**), ROS production (**B**), total thiols content (**C**), and mitochondrial membrane depolarization (**D**) of PBMC incubated with serum collected before and immediately after high-intensity interval exercise (HIIE) or exhaustive exercise. (**E**) is a representative histogram of mitochondrial membrane potential of PBMC incubated with serum collected before and after each exercise protocol. Statistical analysis conducted by two-way repeated measurements ANOVA with Bonferroni’s post hoc (*p* < 0.05).

Both post-exercise serums collected post-HIIE and post-exhaustive exercise protocols changed the PBMC redox state of resting obese individuals. The in vitro treatment of PBMCs from obese individuals with serum collected after HIIE or exhaustive exercise-induced significant lipid peroxidation (*p* = 0.01, ES = 1.97 for interval exercise; *p* = 0.01, ES = 3.91 for exhaustive exercise; Fig. 2A) and reactive oxygen species (ROS) production (*p* = 0.03, ES = 2.06 for HIIE; *p* = 0.02, ES = 5.03 for exhaustive exercise; Fig. 2B) without changes in total thiol content of treated cells (*p* > 0.05; Fig. 2C). The serum from post-exhaustive exercised men induced higher PBMC ROS production compared to serum from post-HIIE exercised men (*p* = 0.04; ES = 1.93; Fig. 2B). Furthermore, mitochondrial depolarization was higher in cells incubated with serum acquired from post-exhaustive exercised men (*p* = 0.04; ES = 2.0; Fig. 2D).

**Figure 3:**
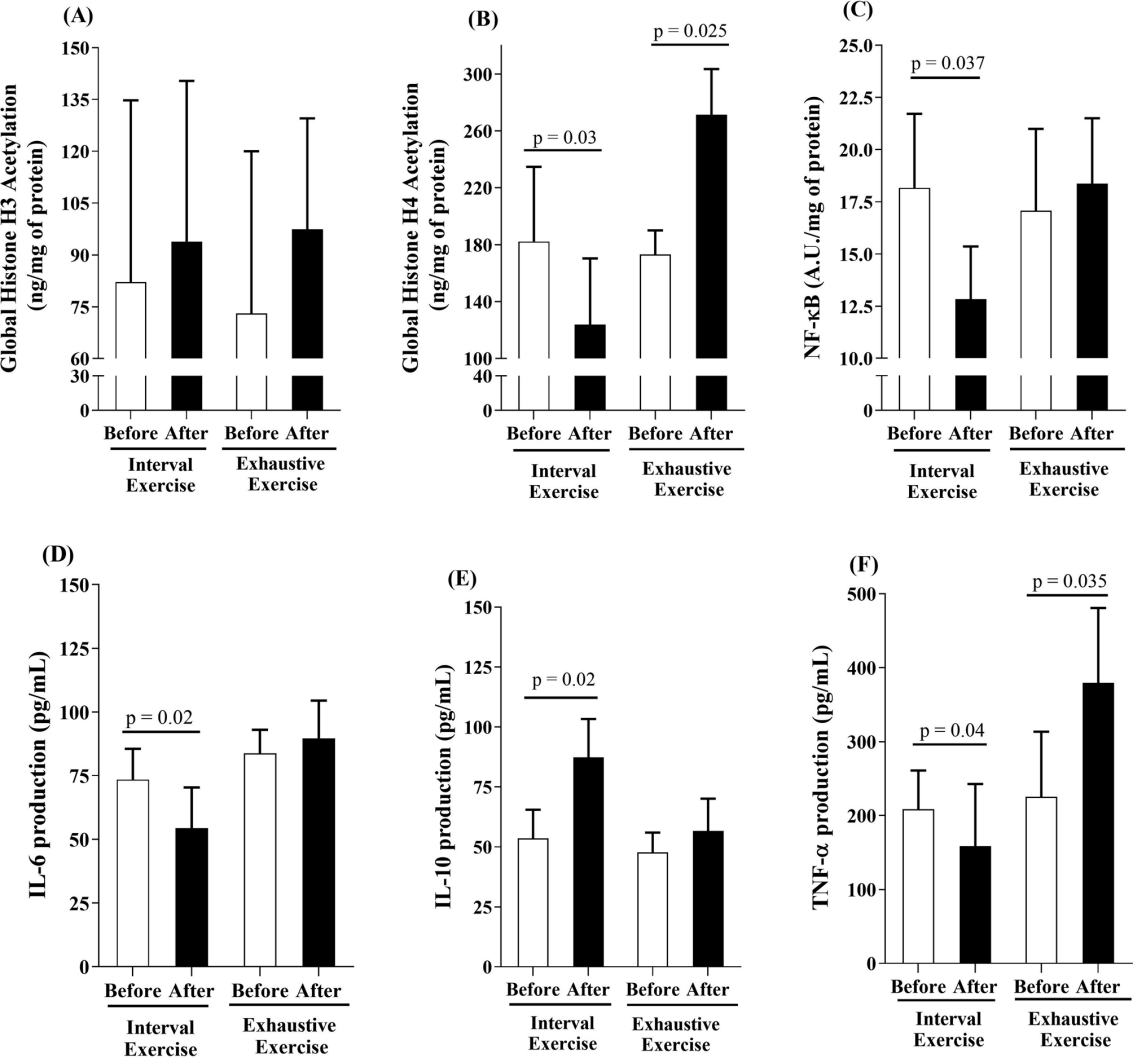
Epigenetic markers, NF-κB content and cytokine production in peripheral blood mononuclear cells incubated in vitro with autologous serum from before (white bars) and after (black bars) two types of exercise. Intracellular content of global H3ac (**2A**), H4ac (**2B**), Nuclear factor kappa B (**2C**) in PBMC, and the production of IL-6 (**2D**), IL-10 (**2E**) and TNF-α (**2F**) by PBMC incubated with serum collected before and immediately after high-intensity interval exercise (HIIE) or exhaustive exercise. Statistical analysis conducted by two-way repeated measurements ANOVA with Bonferroni’s post hoc (*p* < 0.05).

Incubation of PBMCs of resting obese individuals with serum collected post-HIIE decreased the levels of global H4ac (*p* = 0.03; ES = 1.18, Fig. 3B), while treatment with serum from the exhaustive exercise session increased H4ac (*p* = 0.025; ES = 3.83). However, no differences were identified in H3ac after incubation with post-exercise serum of HIIE or exhaustive exercise bouts (*p* > 0.05; Fig. 3A). In addition, the post-HIIE serum decreased the NF-κB p65 total content in nuclear fractions of PBMCs of obese men (*p* = 0.03; ES = 1.73), while there were no changes in post-exhaustive exercise serum treatment (*p* > 0.05) (Fig. 3C). Incubation of PBMCs with post-HIIE serum decreased IL-6 (*p* = 0.02, ES = 1.35; Fig. 3D) and TNF-α (*p* = 0.04, ES = 0.71; Fig. 3F) secretion. In contrast, incubation of PBMCs with serum collected post-exhaustive exercise increased TNF-α production (*p* = 0.03, ES = 1.62; Fig. 3F). Moreover, higher IL-10 production was found in PBMCs stimulated with post-HIIE serum (*p* = 0.02; ES = 2.40; Fig. 3E), with no differences in post-exhaustive exercise serum incubation (*p* > 0.05).

### Autologous serum collected after exercise bouts modulate the frequency of CD4+ T cells expressing CD39, CD73, CTLA-4, and PD-1

**Figure 4:**
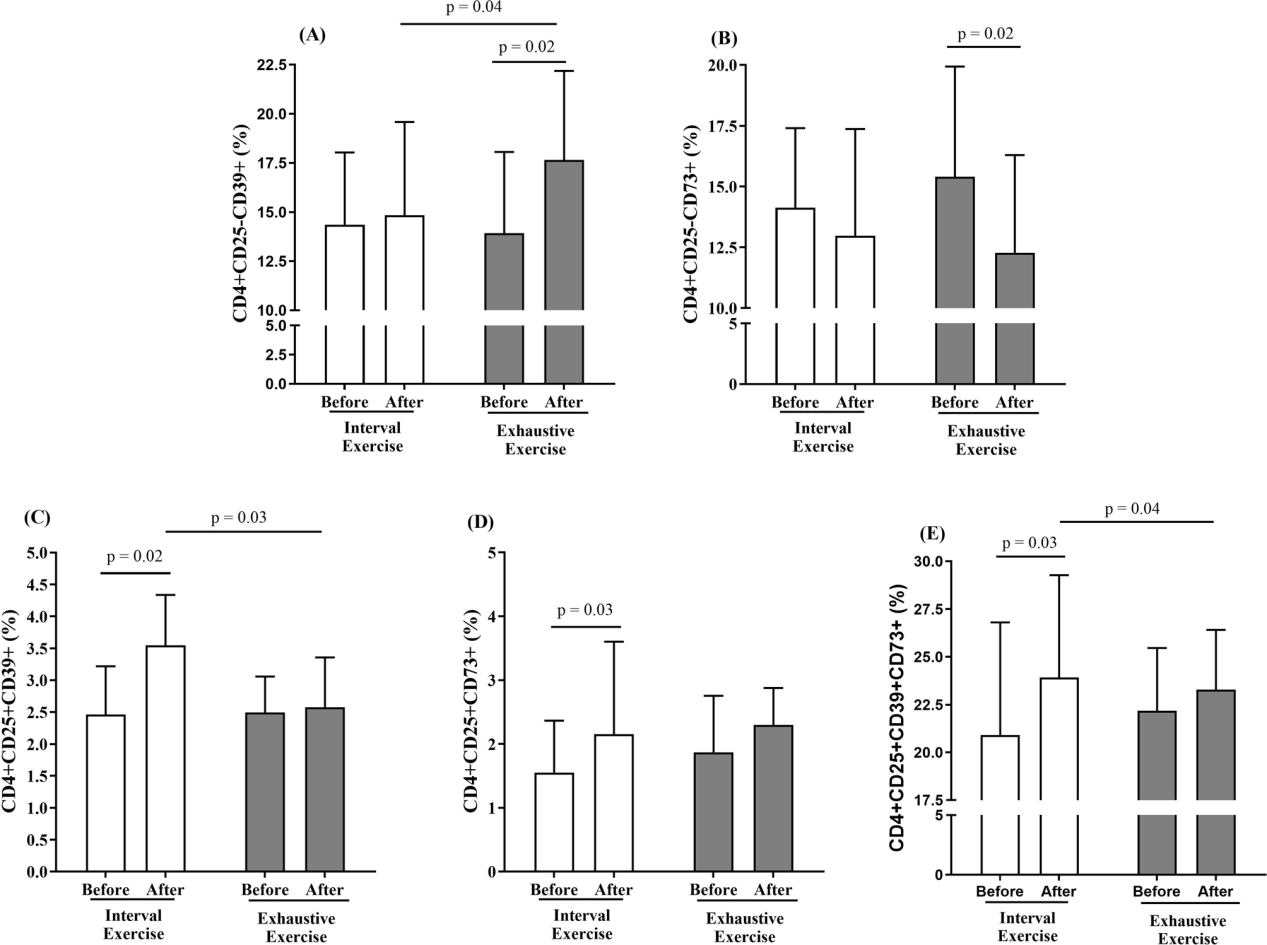
CD4+ T cells expressing CD39 and CD73 ectonucleotidases following in vitro incubation with autologous serum from before (white bars) and after (black bars) two types of exercise. The frequencies of CD4 + CD25-CD39 + (**3A**), CD4 + CD25-CD73 + (**3B**), CD4 + CD25 + CD39 + (**3C**), CD4 + CD25 + CD73 + (**3D**), CD4 + CD25 + CD39 + CD73 + (**3E**) T cells were analyzed by flow cytometry after in vitro PBMC stimulation with serum collected before and immediately after high-intensity interval exercise (HIIE) or exhaustive exercise. Statistical analysis conducted by two-way repeated measurements ANOVA with Bonferroni’s post hoc (*p* < 0.05).

As PBMCs are composed mainly of lymphocytes (approximately 85%), and T cells are more responsive than B cells to exercise, we analyzed the effects of autologous serum from before and after the two types of exercise on CD4 + and CD8 + T cell expressing regulatory molecules (Fig. 4). Autologous post-HIIE serum increased the frequencies of CD4 + CD25 + CD39 + (*p* = 0.02, ES = 1.40; Fig. 4C), CD4 + CD25 + CD73 + (*p* = 0.03, ES = 0.51; Fig. 4D), and CD4 + CD25 + CD39 + CD73+ T cells (*p* = 0.03, ES = 0.54; Fig. 4E) compared to the incubation model with pre-HIIE serum. Conversely, incubation of lymphocytes with serum acquired post-exhaustive exercise increased the frequency of CD4 + CD25-CD39 + (*p* = 0.02, ES = 0.86; Fig. 4A), but decreased the percentage of CD4 + CD25-CD73 + (*p* = 0.02, ES = 0.73; Fig. 4B). CD4 + CD25-CD39+ T cells were higher in post-exhaustive exercise serum incubation compared to post-HIIE serum incubation (*p* = 0.04, ES = 0.61; Fig. 4A). In addition, the frequencies of CD4 + CD25 + CD39 + (*p* = 0.03, ES = 1.24; Fig. 4C) and CD4 + CD25 + CD39 + CD73+ T cells (*p* = 0.04, ES = 0.15; Fig. 4E) were higher in PBMCs incubated with post-HIIE serum in relation to the post-exhaustive exercise serum incubation model.

### Autologous serum collected after exercise bouts modulates the frequency of CD4 + CTLA-4 + and CD8 + PD-1+ T cells

**Figure 5:**
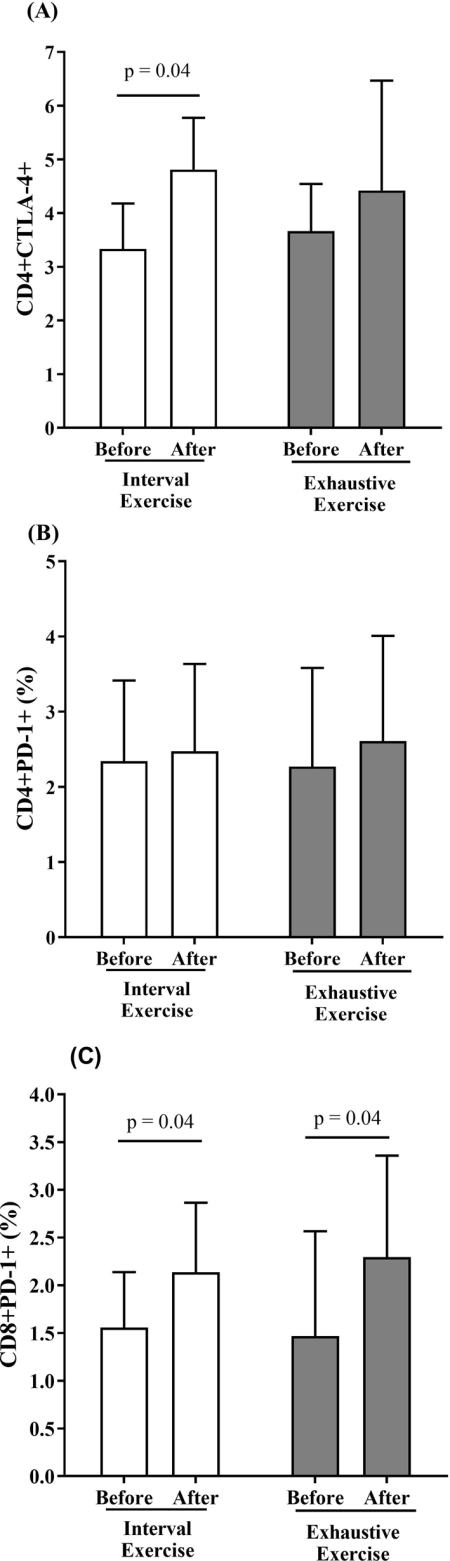
CD4 + and CD8+ T cells expressing PD-1 or CTLA-4 following in vitro incubation with autologous serum from before (white bars) and after (black bars) two types of exercise. The frequencies of CD4 + CTLA-4 + (**4A**), CD4 + PD-1 + (**4B**) and CD8 + PD-1 + (**4C**) T cells were analyzed by flow cytometry after in vitro PBMC stimulation with serum collected before and immediately after high-intensity interval exercise (HIIE) or exhaustive exercise. Statistical analysis conducted by two-way repeated measurements ANOVA with Bonferroni’s post hoc (*p* < 0.05).

We also evaluated the effect of in vitro incubation of PBMCs with post-exercise serum on CD4 + and CD8+ T cells expressing the inhibitory molecules CTLA-4 (in CD4+ T cells) and PD-1 (in CD4 + and CD8+ T cells). Lymphocytes incubated with autologous serum collected post-HIIE showed an increased frequency of CD4 + CTLA-4 + (*p* = 0.04, ES = 1.64; Fig. 5A) compared to the incubation model with serum collected pre-HIIE. The frequency of CD8 + PD-1+ T cells was higher in both post-exercise serum incubation models (HIIE: *p* = 0.04, ES = 0.89; exhaustive exercise: *p* = 0.04, ES = 0.77; Fig. 5C). No statistical difference was found in CD4 + PD-1+ T cells in both groups (Fig. 5B; *p* > 0.05).

## Discussion

This report provides the first evidence that systemic factors released into the peripheral blood during different types of exercise bouts differentially change several parameters related to the immune function in the resting PBMCs of obese men. We observed that the inflammatory response and lymphocyte phenotypical markers in PBMCs incubated with post-exercise serum were dependent on the exercise type: (a) post-HIIE serum incubation increased the frequencies of CD4 + CD25 + CD39 +, CD4 + CD25 + CD73 +, CD4 + CD25 + CD39 + CD73 +, CD4 + CTLA-4 +, and CD8 + PD-1+ T cells and the production of IL-10 concomitant to the decreased NF-κB content, H4ac levels, TNF-α, and IL-6 production in PBMCs and (b) higher TNF-α production and H4ac levels, increased frequencies of CD4 + CD25-CD39 + and CD8 + PD-1+ T cells, and lower proportions of CD4 + CD25-CD73+ T cells occurred after the incubation of PBMCs with autologous serum collected after exhaustive exercise. Higher lipid peroxidation and ROS production were observed in PBMCs incubated with serum collected after both HIIE and exhaustive exercise. Moreover, the incubation of PBMCs with serum collected after the exhaustive bout led to significant mitochondrial membrane depolarization. Thus, the changes in the PBMC parameters were dependent on the type of the exercise bout, since opposite effects were observed on the H4ac levels, inflammatory response, and T cell phenotype in serum obtained after HIIE and exhaustive exercise.

First, we evaluated some systemic biochemical and immunological parameters in the same obese men who were subjected to an acute HIIE session and an acute exhaustive exercise session. The systemic and physiological responses to exercise are complex and not well understood but involve a wide range of metabolic, immunological, and neuroendocrine changes^24^. Here, we analyzed serum biomarkers that are commonly modulated by acute exercise, including cytokines (IL-6, IL-10, IL-17a, IFN-γ, and TNF-α), adipokines (leptin), blood hormones (cortisol and IGF-1), metabolic molecules (glucose and lactate), and oxidative stress markers (TBARS and uric acid). Changes in these above-mentioned parameters are dependent on the type, volume, and intensity of the bout. A similar increase in the systemic levels of IL-6, IGF-1, cortisol, lactate, uric acid, and TBARS were observed after both HIIE and exhaustive exercise. However, only HIIE induced a significant increase in IL-10 plasma levels and decrease in leptin concentrations. Exhaustive exercise enhanced the systemic TNF-α and IL-17a serum concentrations. These results denote that while both exercise protocols lead to systemic metabolic perturbations, the cytokine response is dependent on the type and mode of the exercise protocol. In fact, we and others demonstrated the role of HIIE in the increase of systemic IL-10 and other immunoregulatory cytokines, such as IL-1Ra and TGF-β, in a variety of subjects^40–43^. In addition, de Souza et al. also identified lower levels of leptin after interval exercise, which may indicate an anti-inflammatory effect of exercise by a decrease in the release of adipokine from adipose tissue^41^. Conversely, the strong pro-inflammatory response may be linked to the higher muscle damage degree observed in exhaustive exercise^44^. Suzuki and coworkers^45^ found increased concentrations of systemic anti-inflammatory (IL-1ra and IL-10) and pro-inflammatory (IL-8) cytokines, associated with rises in hormones (cortisol and GH) and markers of muscle damage in the serum of athletes after a marathon race. The increases in the concentrations of immunoregulatory cytokines after strenuous exercise may be an attempt to counter the uncontrolled inflammatory response induced by continuous hormone stimulation and increased muscle damage after the bout.

It is well known that the systemic physiological perturbations, such as microbial translocation, muscle damage, sympathetic discharge, immunometabolism, and increased myokine expression, can directly alter the leukocyte phenotype and function during and after exercise^4,22–24,46^. Previous studies examined the immunomodulatory effects of serum obtained after endurance exercise on PBMCs^22,23^. PBMCs treated with 50% autologous post-exercise serum for 18 h increased HLA-DR expression in total monocytes and CD14 + CD16 + pro-inflammatory monocytes^23^. Moreover, PBMCs from healthy men incubated with post-exercise serum obtained after 1 h of exercise increased NK cytotoxic activity, which inversely correlated with changes in serum cortisol levels after the bout^22^.

In this sense, we believed that systemic factors released into peripheral blood during exercise may directly influence immune function and the T cell phenotype of resting PBMCs. Our study hypotheses were partially confirmed. Here, we observed that acute HIIE induces significant mobilization of CD4 + CD25-CD39 +, CD4 + CD25 + CD39 +, CD4 + CD25 + CD73 +, CD4 + CTLA-4 +, CD4 + PD-1 + and CD8 + PD-1+ T cells into peripheral blood. Interestingly, most of these T cell subsets changed in our in vitro PBMC incubation with post-exercise autologous serum. In this regard, in vitro PBMC incubation with post-HIIE autologous serum increased the frequencies of CD4+ T cells expressing CD39/CD73 ectonucleotidases, CTLA-4, and PD-1, which may represent the immunoregulatory effects of HIIE in obese individuals. In fact, all these aforementioned molecules are now recognized as immune checkpoints that hamper the inflammatory response^47,48^. The CD39/CD73 axis metabolizes ATP to adenosine, and downregulates NF-κB expression in immune cells^13^. In addition, CTLA-4 exerts cell-to-cell immunosuppressive actions by interacting with CD80/CD86 of antigen-presenting cells (APCs), thus inhibiting the induction of the adaptive immune response by lowering CD28 expression^14,49^. Regarding the ectonucleotidases, we previously showed that HIIE increases the frequencies of CD4 + CD25 + CD39 + CD73+ T cells, a memory Treg (mTreg) phenotype, in sedentary and highly active men^6^.

Indeed, PD-1 is a key tolerogenic T cell molecule that is highly expressed on memory T cells and involved in modulating cell activation, differentiation, and migration to inflamed tissues by dampening T cell receptor (TCR) signaling and decreasing T cell functions, such as cytokine production, T cell proliferation, and cell migration^15,50^. Gustafsson et al.^51^ recently reported changes in peripheral CD4 + and CD8+ T cells expressing PD-1 after 45 min of maximal cycling exercise. Furthermore, HIIE seems to be superior in increasing the peripheral proportions of central memory and effector memory CD8+ T cells expressing PD-1 than moderate continuous exercise. The increased PD-1 expression on these cells after HIIE may be involved in the maintenance of homeostasis, decrease in the activation, differentiation, and redistribution of less differentiated memory phenotypes, such as central memory T cells, in a physiological condition^52^. Thus, HIIE mobilizes T cells expressing checkpoints that may be linked to the anti-inflammatory effects of exercise. Our data highlight the effect of exercise-induced blood factors on the upregulation of immune checkpoint molecules that can have an impact on the inflammatory profile observed in obese individuals.

Although exercise modifies a milieu of molecules in the blood that alter immune function, we identified a significant decrease in systemic leptin levels concomitant with higher lactate levels after HIIE. Leptin activates T cells and reduces the Treg phenotype as well as CTLA-4 expression by stimulating downstream pathways, phosphatidylinositol-3 kinase (PI3K), and protein kinase A (PKA)^53,54^. Moreover, the synthesis and expression of PD-1 by memory CD4 + and CD8+ T cells are stimulated by higher glycolytic activity and lactate consumption by T cells^55^. Hence, metabolic changes in T cells may be induced by blood factors released during exercise that together may favor a homeostatic status. Circulating lactate concentrations increase during exercise, and exhaustive exercise activities can rise to 100-fold on active muscles, with a concomitant increase of more than tenfold in blood serum^56^. Moreover, PBMC incubation with lactate at moderate-intensity concentrations (3.5 mM) blunted the LPS-induced pro-inflammatory cytokine production^35^. However, exercise induces the secretion of a wide range of metabolic products that could affect the immune function, and in vitro PBMC incubation with post-exercise serum does not allow for analyzing the individual effect of each molecule found in the serum on leukocyte function and phenotype.

The in vitro incubation of PBMCs with autologous serum obtained after exhaustive exercise increased the frequencies of CD4 + CD25-CD39 + memory effector T cells (mTeff) and CD8 + PD-1+ T cells, and decreased the frequency of CD4 + CD25-CD73+ T cells. Conversely, the peripheral blood frequencies of CD4 + CD25-CD39 +, CD4 + PD-1 +, and CD8 + PD-1+ T cells increased after exhaustive exercise, concomitant with a decrease in the proportions of CD4 + CD25-CD73 +, CD4 + CD25 + CD73 +, and CD4 + CTLA-4+ T cells. Therefore, changes in blood factor composition after exhaustive exercise may not explain all T cell phenotype changes observed after the bout.

Interestingly, CD39 expression is a marker of T cell activation, and CD4 + CD25-CD39 + mTeff cells produce higher levels of IL-17a and IFN-γ^57^. The CD4 + CD25-CD39 + T cell phenotype presents signs of metabolic stress (i.e. alterations in redox state) and high susceptibility to apoptosis^58^, and these cells were previously related to lower immune tolerance^59^. It was previously reported that exhaustive exercise leads to higher CD4 + Th17 phenotype and lowered Treg counts in the peripheral blood of highly trained men^60^. Furthermore, in vitro incubation of resting PBMCs with post-exhaustive exercise serum in a study by Perry et al.^60^ led to decrease in CD4 + CD25 + FoxP3 + Treg frequency. Thus, the higher systemic stress induced by exhaustive exercise may induce a strong inflammatory response through the induction of CD25-CD39 + phenotype and lower CTLA-4 expression in CD4+ T cells. Moreover, the decreased CD4 + CD25-CD73 + T cell proportions in PBMCs incubated with post-exhaustive exercise autologous serum may be linked with a hampered ability to metabolize ATP by CD4+ T cells.

Here, we shed light on the exercise-induced epigenetic changes in immune cells mediated by systemic factors released into the peripheral blood. The observations that a differential response of global H4ac in PBMCs stimulated with post-exercise serum from HIIE or exhaustive exercise are in line with previous studies that demonstrate that exercise changes epigenetic machinery in immune cells^9,61^. Our data revealed that the incubation of post-HIIE serum reduced the H4ac status and NF-κB content in the nucleus of PBMCs. In fact, data from our group demonstrated that an acute bout of HIIE increased the intracellular global histone deacetylase (HDAC) activity in PBMCs, which is responsible for removing the acetyl group from the N-terminal tail of the lysine site of histones, in association with higher plasma IL-10 and TGF-β concentrations^62^. The decreased NF-κB content in the nucleus of PBMCs identified after the incubation with post-HIIE serum may be related to the downregulation of Toll-like receptors (TLR) on the cell surface of lymphocytes and monocytes^63^. Furthermore, acute high-intensity exercise releases pro-resolving lipid mediators such as resolvins and maresins into peripheral blood that contribute to the resolution of inflammation through receptor-mediated actions on leukocytes^64^. Exposure of naïve bone marrow-derived macrophages to plasma isolated from exercised mice promotes the resolution of acute inflammation by stimulating macrophage phagocytosis and lowering inflammatory cytokine expression through enhanced pro-resolving pathways^65^. Incubation of Jurkat cells with 10% of post-high-intensity exercise (80% of maximal work rate) serum decreased TNF-α production^34^. Collectively, these results may indicate that serum factors released during HIIE induce a condensed state of chromatin and lower NF-κB activity to inflammatory gene transcription in peripheral leukocytes.

The increases in IL-10 production by PBMCs stimulated with post-HIIE serum are in line with the immunoregulatory phenotype associated with the higher proportions of CD4+ T cells expressing CD39/CD73 and CTLA-4 induced by post-exercise serum factors. IL-10 blunts IκB kinase and NF-κB DNA binding activity, suppressing the pro-inflammatory gene expression^66^. In contrast, serum collected post-exhaustive exercise increased TNF-α production and the H4ac status of PBMCs. In fact, the higher muscle damage, systemic endotoxin levels, and ROS generation were associated with increased TLR expression on monocyte cell surface and NF-κB phosphorylation after exhaustive exercise^67^. In a rat model study, Goutianos et al^36^ showed that intravenous administration of plasma collected from exhaustive exercise trained rats increased inflammatory cytokine expression (IL-2, IL-6, IL-8, TNF-α, and C-reactive protein) measured in plasma samples, skeletal muscle, and adipose tissue of non-exercised rats. We previously demonstrated that exhaustive exercise-induced a state of histone H4 hyperacetylation status and increased TNF-α and IL-8 production by LPS-stimulated PBMCs in obese individuals^9^. Thus, exhaustive exercise induces the release of both danger and pathogen associated molecular patterns to the peripheral blood that are recognized by TLRs and other pattern recognition receptors that increase the intracellular inflammatory signaling cascade in mononuclear cells.

Here, we found similar changes in redox states in PBMCs after exposure to serum collected after HIIE or exhaustive exercise, as observed by higher ROS production and TBARS content in the cell lysate. Higher levels of lipid peroxidation observed in PBMCs after exercise are related to increased metabolic process and mitochondrial respiration of these cells during the bout^68–71^. In contrast, the incubation of zymosan-stimulated phagocytes with post-marathon race plasma leads to decreased ROS production^33^. This contradictory finding may be related to the some experimental conditions in the study of Katsuhiko et al^33^, such as the antigenic stimulation of myeloid cells (monocytes and granulocytes) and the highly trained study population. In fact, it was suggested that biochemical adaptations in the athletic population induce higher antioxidant protection, which can quickly control acute ROS elevations after exhausting exercise^72^. On the other hand, obese individuals present low antioxidant enzymes in both serum and PBMC, and maximal exercise leads to a higher imbalance in the redox state in lymphocytes of obeses^73,74^. Therefore, in our study it is possible that ROS elevation in PBMC incubated with post-exercise serum may be related to lower systemic antioxidant mediators in the serum of obese individuals after the bouts. The thiobarbituric acid (TBA) reactivity test is a reliable estimator for identifying several end products formed through the decomposition of lipid peroxidation products^75^. However, a variety of other lipid compounds, such as oxidized lipids and saturated/unsaturated aldehydes, interfere in the assay causing overestimation of malondialdehyde (MDA) concentration^76^. In this respect, we recognize that TBARS analysis is one of the main limitations of our study for its low sensitivity and selectivity since many MDA-unrelated molecules can react with TBA, and some artifactual generation of MDA during the assay has been raised^75^.

In addition, post-exhaustive exercise serum incubation leads to significant increase in mitochondrial membrane depolarization of lymphocytes. Mitochondrial membrane depolarization leads to cytochrome c leakage and precedes the activation of caspases that induces degradation of several proteins, and is involved in events of cell death, such as DNA fragmentation and apoptosis^77–79^. Hence, the increased TNF-α, mitochondrial membrane depolarization, and senescent T cell phenotype induced by autologous post-exhaustive exercise serum stimulation may be associated with the induction of cell death by systemic mediators released during the bout into peripheral circulation.

This study demonstrated that systemic factors released in response to exercise modify the PBMC H4ac status, NF-κB levels in the nucleus of PBMCs, cytokine production and CD4 + and CD8+ T cells expressing ectonucleotidases, CTLA-4, and PD-1. Although the precise signaling systemic molecules involved in post-translational histone acetylation remain to be elucidated, our data suggest that a differential response to HIIE and exhaustive exercise may alter the immune and epigenetic status of PBMCs. Our study hypothesis was partially confirmed, and the effects of the two exercise types on the immune parameters analyzed in the peripheral blood, and after the in vitro incubation of resting PBMCs with autologous serum collected before and after exercise bouts are qualitatively summarized in Table 3. Collectively, the data suggests an influence of systemic factors released during exercise in the immunomodulatory effects promoted by exercise.

**Table 3:** Summary of the differences between peripheral blood immune response and in vitro experiments with the autologous serum of obese men acquired after two exercise sessions.

	Acute HIIE		Acute exhaustive exercise	
	Peripheral blood	In vitro post-exercise autologous serum incubation	Peripheral blood	In vitro post-exercise autologous serum incubation
***Inflammatory markers***				
IL-6	↑	↓	↑	↔
IL-10	↑	↑	↔	↔
TNF-α	↔	↓	↑	↑
NF-κB	–	↓	–	↔
***Redox state***				
TBARS	↑	↑	↑	↑
ROS production	–	↑	–	↑
MMP	–	↔	–	↑
***Epigenetic markers***				
H4Ac	–	↓	–	↑
***T-cell phenotype***				
CD4 + CD25-CD39 +	↑	↔	↑	↑
CD4 + CD25-CD73 +	↔	↔	↓	↓
CD4 + CD25 + CD39 +	↑	↑	↔	↔
CD4 + CD25 + CD73 +	↑	↑	↓	↔
CD4 + CD25 + CD39 + CD73 +	↔	↑	↔	↔
CD4 + CTLA-4 +	↑	↑	↓	↔
CD4 + PD-1 +	↑	↔	↑	↔
CD8 + PD-1 +	↑	↑	↑	↑

↔, unchanged; ↑, increased; ↓, decreased; IL, interleukin; NF-κB, Nuclear Factor-kappaB; TNF-α, Tumor necrosis factor-alpha; TBARS, Thiobarbituric acid reactive substances; MMP, mitochondrial membrane potential; H4Ac, Histone H4 acetylation.

## Methods

### Ethical statements and participants

This study was approved by the Research Ethics Committee of the Universidade Federal de Ciências da Saúde de Porto Alegre (protocol 1.973.432) and all experimental procedures were performed according to the Declaration of Helsinki. All study subjects were informed about the study and signed the informed consent form. Eight sedentary normoglycemic obese men (age 28.42 ± 4.19 years; height 1.75 ± 0.03 m; body mass 99.8 ± 3.5 kg; body mass index 32.81 ± 2.19 kg/m^2^; waist circumference 105.12 ± 8.14 cm; body fat 32.2 ± 3.9%; fasting glucose 88.29 ± 9.21 mg/dL; maximal oxygen consumption 40.12 ± 4.28 mL/kg/min) participated in this study. Exclusion criteria included autoimmune, cardiac, endocrine or metabolic diseases, acute and chronic infections, and under current pharmacological drugs known to affect the metabolic (i.e., hypoglycemic drugs, statins, and other lipid-lowering drugs) or immune system (i.e., steroidal and non-steroidal anti-inflammatory drugs and immunomodulatory medications), or dietary supplements with recognized impact on the immune system.

### Preliminary trial

One week before the experimental trial, all participants went to the Human Movement Laboratory of UFCSPA (Porto Alegre/Brazil) to estimate their body composition and cardiorespiratory fitness. Body mass (kg) and height (meters) were determined by a semi-analytical scale with a stadiometer attached (Welmy, Santa Barbara D’Oeste, Brazil). Waist circumferences (WC, cm) were measured through an inelastic measuring tape. Body fat percent was measured as previously reported^9^. The cardiopulmonary exercise testing was performed to determine the intensity of exercise sessions using a ramp protocol^80^. The exercise test started at 4.5 km/h, with no slope for all study participants. Then, the load (speed and slope) was individually increased for each participant considering their physical condition and tolerance to obtain maximal oxygen consumption (VO_2Max_) within 8–12 min. The test was terminated after verification of the following circumstances: (a) request of the participant due to extreme tiredness and/or perception of the intense sensation of dyspnea; (b) attained the maximum heart rate (HR) predicted by age (HRmax) ≥ 85%; (c) peak respiratory exchange ratio RER > 1.1; (d) VO2 plateau was reached even with increasing workload. Ventilatory and metabolic parameters were collected by respiration using Metalyzer 3B (Cortex, Leipzig, Germany), and were analyzed after the mean of the data in eight respiratory cycles. The VO2 values were analyzed with averages of the last 10 s of each stage and the VO2_Max_ was assumed as the stabilization of the highest average value attained during the test, while the highest sustained velocity during the entire stage was assumed to be MAV.

### Main trials and blood collection

All subjects were subjected to both protocols (HIIE and exhaustive exercise) with an interval of at least one week between sessions. Both trials were conducted in the Human Movement Laboratory of UFCSPA (Porto Alegre/Brazil). Blood samples (15 mL) were collected before and immediately after each exercise session (pre and post each bout) into tubes without anticoagulant, and with ethylenediaminetetraacetic acid (EDTA). Tubes without anticoagulant were centrifuged (2000* g,* 10 min) and the serum was aliquoted and frozen (-80ºC) for cell culture experiments. The HIIE session consisted of a warm‐up (5 min) at a workload eliciting 40% HRmax. The HIIE consisted of 10 bouts of 60 s (85–90% MAV) alternated with 75 s of recovery (50% MAV) on a motorized treadmill^40^. The exhaustive exercise consisted of stepping up and down from a step adjusted to the height of each subject’s femoral condyles^9,81^. The stepping rhythm was paced acoustically at 60 beats per min with the same periods (1 s) for stepping up and down, respectively. When the participants were not able to maintain the required stepping rhythm, a 30 s recovery period was given.

During exercise, gas analysis data were monitored online using Metalyzer 3B (Cortex, Leipzig, Germany), and HR by telemetry. We equalized the exercise sessions by the energy expenditure (EE) of HIIE. Thus, the EE of exhaustive exercise should be equal to those achieved during HIIE. The EE during exercise was calculated based on the metabolic equivalent method^82^. The EE was calculated at the end of every minute in both sessions, and exhaustive exercise stopped once the target EE had been attained.

### Biochemical analysis of the peripheral blood

Biochemical analysis was conducted in the serum from the participants collected before and after each exercise bout. Systemic leptin (Peprotech Inc, USA) and insulin growth factor-1 (IGF-1) (BOSTER, USA) levels were evaluated by enzyme-linked immunosorbent assay (ELISA). Cortisol concentrations were measured using an Immulite immunoassay system (Siemens, USA). Blood glucose, lactate, and uric acid levels were evaluated through automated biochemical assay using Bioclin BS120 (BioClin, Brazil).

### PBMC isolation and stimulation

The PBMCs of each participant was isolated before and after each exercise bout. Briefly, PBMCs were isolated from peripheral blood from participants using histopaque gradient solution, Histopaque 1077 (Sigma‐Aldrich, St Louis, MO, USA) as previously described^9^. PBMC viability was determined by trypan blue exclusion and the viability was always more than 95%. Then, the cells were washed and suspended in Roswell Park Memorial Institute‐1640 medium (Sigma‐Aldrich, USA) supplemented with 2 g/L sodium bicarbonate, 2% glutamine, and 100 U/mL penicillin – 0.1 mg/mL streptomycin (Sigma‐Aldrich, USA).

To determine the contribution of exercise-induced systemic mediators on lymphocyte phenotype and inflammatory profile, PBMCs from each participant acquired pre-exercise condition were incubated with 50% of their own serum collected before and immediately after each exercise protocol. This concentration of serum approximates to the proportion of serum in human blood and has been used in other works^22,23^. Cells (1 × 10^6^/mL) were incubated in RPMI-1640 with 50% of serum collected before and after HIIE and exhaustive exercise bout for 4 h (5% CO_2_, 37 °C) in 24-well plates in a final volume of 1 mL. Then, the culture supernatants were collected, centrifuged (400 g, 25 °C, 10 min), and kept at − 80 °C for cytokine quantification. PBMCs were collected, washed with phosphate-saline buffer 1x, and frozen at − 80 °C in a solution containing 90% fetal bovine serum and 10% dimethyl sulfoxide (DMSO) for epigenetic analysis. For T cell phenotyping, PBMCs were collected, immediately stained with antibody-fluorochrome, and analyzed with flow cytometry.

### Histone H3 and H4 acetylation status and NF-κB activity in PBMCs

Histone extracts from PBMCs were prepared using a total histone extraction kit (EpiGentek, USA) according to the manufacturer’s protocol. H4ac and H3ac levels in PBMCs were determined using the Global Histone H4 and Global Histone H3 Acetylation Assay Kits (Colorimetric Detection, EpiQuik, USA) according to the manufacturer’s instructions and expressed as ng/mg protein. The nuclear fraction of the isolated PBMCs was extracted using a commercially available nuclear extraction kit (Epiquik Nuclear Extraction Kit, USA) according to the manufacturer’s protocol. The levels of total NFκB p65 in the nuclear fraction, which had been isolated from lysed cells by centrifugation, were measured using an ELISA commercial kit from Invitrogen (NFkB p65Total Human InstantOne ELISA Kit, ThermoFisher, USA). The nuclear protein concentration was determined using a Bradford assay^83^, and the activated NF-kB p65 level was expressed as arbitrary units per mg of protein (AU/mg of nuclear protein).

### Assessment of cytokine levels and oxidative stress markers

The concentrations of TNF-α, IL-6, IL-10, IL-17a and interferon-gamma (IFN-γ) (all from eBioscience, USA) in the supernatants of serum-stimulated cells and serum from participants were quantified by ELISA kits, according to the manufacturer’s protocols. Lipid peroxidation was measured through the TBARS in PBMCs following a previously described protocol^69^ and expressed in MDA nmol/mL. Total thiol concentrations were measured in PBMCs using 5–5′-dithio-bis(2-nitrobenzoic acid) reagent^9^. ROS production was evaluation in the cell lysate through the fluorescence intensity of the redox-sensitive dye 2′,7′-dichlorodihydrofluorescein diacetate (DCFH, 100 µM, Sigma-Aldrich) (excitation and emission wavelengths of 480 and 535 nm, respectively) using SpectraMax M2e (Molecular Devices, USA).

### Lymphocyte cell stanning and flow cytometry evaluation

The phenotype of freshly isolated PBMCs and PBMCs cultured in vitro with autologous serum collected before and after exercise were evaluated by flow cytometry. Briefly, 2 × 10^5^ PBMCs were stained with monoclonal antibodies (all anti-human) conjugated with specific fluorochromes: CD4 FITC, CD8 PerCP, CD25 Pe, CD39 PerCP-7, CD73 APC (all from Ebioscience, USA); CD125 (CTLA-4) Pe, CD279 (PD-1) Pe (all from Biogems, USA). Cell phenotype was acquired using CELLQuest Pro Software (BD Bioscience, USA) on a FACSCalibur flow cytometer (BD Bioscience, USA). A minimal of 30,000 events/tubes were acquired, and lymphocyte were identified and gated according to each forward scatter (FSC) and side scatter (SSC) profile. The frequencies of CD4 + CD25-CD39 +, CD4 + CD25-CD73 +, CD4 + CD25 + CD39 +, CD4 + CD25 + CD73 +, CD4 + CD25 + CD39 + CD73 +, CD4 + CTLA-4 +, CD4 + PD-1 +, and CD8 + PD-1+ T cells were evaluated. Single color control tubes and negative control were used in each assay to account for spectral overlap.

The mitochondrial membrane potential (ΔΨm) was quantified according to a method previously described^84^, using the fluorescent dye rhodamine 123 (Rh 123, Sigma-Aldrich). The analysis was performed using a BD FACSCalibur (Becton–Dickinson, Rutherford, NJ, USA) flow cytometer and CellQuest Pro software (Joseph Trotter, Scripps Research Institute, La Jolla, CA, USA), using the blue argon-ion 488 nm laser with the FL1filter channel. A total of 30,000 events were acquired in the region that corresponded to the lymphocytes.

### Statistical analysis

Data were analyzed using SPSS 20.0 (SPSS Inc, IBM, Chicago, IL, USA). After the normality test by Shapiro‐Wilk, all values were presented as mean ± standard deviation (SD). Two‐way repeated measurement analysis of variance (ANOVA), taking time and exercise as factors, were performed followed by Bonferroni’s post hoc for multiple comparisons. *p* ≤ 0.05 was adopted in all analysis. In addition, Cohen’s *d* ES were calculated using G*Power 3.1.9 for significant results.

## Data Availability

The data will be available when requested.
